# To send or not to send: weighing the costs and benefits of mailing an advance letter to participants before a telephone survey

**DOI:** 10.1186/s13104-018-3920-6

**Published:** 2018-11-15

**Authors:** Christina Schell, Alexandra Godinho, Vladyslav Kushnir, John A. Cunningham

**Affiliations:** 1 Institute of Mental Health and Policy Research, Centre for Addiction and Mental Health, 33 Russell St., Toronto, ON M5S 2S1 Canada; 2 St. Michael’s Hospital, Toronto, Canada; 3 Department of Psychiatry, University of Toronto, Toronto, Canada; 4 Austalian National University, Canberra, Australia

**Keywords:** Advance letter, Pre-notification, Telephone survey, Smokers, Smoking cessation, Cost-analysis

## Abstract

**Objective:**

A letter was mailed to half the participants (Letter = 137; No Letter = 138) of a 5-year follow-up survey regarding smoking cessation before attempting contact for a telephone interview. The primary outcome was the number of completed surveys per group (response rate). Secondary analyses of the number of telephone calls placed and a cost analysis were performed.

**Results:**

No conclusive effect was found on the response rates per group (59.1% Letter, 50.0% No Letter; *p *= 0.147). Additionally, a logistic regression, controlling for demographics, revealed that there was no direct effect of sending the letter on response rate (p = 0.369). Non-parametric analysis showed significantly fewer calls (U = 7962.5, z = − 2.274, p < 0.05 two-tailed) and significantly lower costs (U = 11112.00, z = 2.521, p < 0.05 two-tailed) in reaching participants in the Letter group. Mailing an advance letter to participants did not appear to effect response rates between the groups, even when controlling for demographics. However, further analysis examining the number of call attempts and the costs per group revealed the letter may have had other effects. These findings suggest that additional analyses may be merited when evaluating the effectiveness of methods to increase participation, such as an advance letter, especially in cases where the literature largely supports its effectual use.

*Trial registration* ClinicalTrials.gov NCT03097445. Registered 31 March 2017

**Electronic supplementary material:**

The online version of this article (10.1186/s13104-018-3920-6) contains supplementary material, which is available to authorized users.

## Introduction

While the use of questionnaires in research has increased during the last century, participation in survey research continues to decline [1–4]. This has become especially apparent in telephone surveys [2], where cultural norms make it common for individuals to ignore and block unwanted calls [1, 5, 6]. The greater ability and availability of the Internet has also shifted some telephone activities to online platforms [1], further changing telecommunication practices.

Methods to improve response rates in survey research have become paramount; especially given the documented effects on results (increased bias, reduced confidence, statistical power) [7]. The importance of obtaining high response rates has generated considerable effort to improve methods and increase participation. Incentives, questionnaire length, interviewer characteristics, persuasion strategies, and physical characteristics of surveys (e.g. question layout, response options, paper quality) are commonly studied and applied to postal, electronic, and telephone questionnaires [4, 5, 7].

The practice of sending an advance letter to a household, or participant, before attempting telephone contact is particularly common. This method has been investigated since at least the 1970s [8] and continues to be widely used. Mailed letters are often viewed as relatively easy and cost-effective. Furthermore, advance letters can legitimize a study, dispel suspicion or surprise from a ‘cold call’, and create feelings of social exchange [8].

The effectiveness of advance letters has been summarized by de Leeuw et al. [8] and more recently by vanGelder et al. [7] who focused on advance letter use in observational, health-related studies. Both meta-analyses show an improvement in response rates through sending an advance letter, however, they also note considerable heterogeneity in the studies analyzed, which may suggest variation in the intervention effects. This is supported by other studies in the literature that do not report any effect of mailing an advance letter [9, 10]. In an attempt to further the evidence-base regarding the use of advance letters, this work assesses the effect of mailing an advance letter to a sample of smokers before attempting to conduct a 5-year follow-up by telephone subsequent to a period of noncontact. In particular, we wish to address whether advance letters effect response rates (proportion of completed interviews of all eligible participants), resource use, and cost-effectiveness associated with this strategy.

## Main text

### Methods

#### Participants

This project was an extension of a previously reported randomized clinical trial (RCT) [11, 12]. In the original study, 999 Canadian smokers who were interested in receiving free mailed nicotine replacement therapy (NRT) were recruited via random-digit dialing between June 2012, and June 2014. Eligibility criteria included being 18+ years old, smoking more than 10 cigarettes per day, having no contra-indications to NRT, being willing to be followed-up (8-weeks and 6-months), and providing saliva samples. Five-years following recruitment, participants who indicated their willingness to be contacted for future research (time to next contact not specified), or were lost-to-follow-up, were eligible to be re-contacted (N = 924).

#### Procedures

The purpose of the survey was to evaluate the long-term effectiveness of mailed NRT to smokers [13]. Interviewing began June 4, 2017, and participants were contacted using the rigorous call schedule described in the protocol [13].

In an attempt to increase survey completion rates, an advance letter was added to the study protocol, following ethics approval, approximately 4 months after follow-up interviews began. The mailed letter informed participants they would be contacted by telephone and be asked to participate in a research study (see Sample Advance Letter, Additional file 1). Letters were sent 2 weeks before the first call attempt to participants due to be interviewed after September 24, 2017. Participants who were due before this date were not sent a letter retrospectively.

Of the participants eligible to participate, 140 interviews were scheduled between June 4 and September 24, 2017 (No Letter group). Using an equivalent 113-day window, a second comparison group was created, consisting of 139 participants whose interview was scheduled between September 25, 2017, and January 15, 2018. All participants in the second group were sent an advance letter (Letter group). If any participant was not reached by telephone, an Internet search of public records and phone directories was performed [13], which determined four participants were deceased (two per group; removed from the analysis). Thus, the final sample size was 138 participants in the No Letter group and 137 participants in the Letter group.

#### Outcomes

The primary outcome was the difference in response rate between the Letter and No Letter groups and was defined as the proportion of all completed interviews of all eligible participants [14]. Two secondary outcomes were also investigated. Resource use was measured as the number of call attempts made to each participant as this represented the time and labour of placing and logging calls by the interviewer. Finally, the cost of preparing and sending the advance letter, in addition to the cost per telephone station hour, were used in the cost-analysis.

#### Data analysis plan

Although the current project is a follow-up, the analyses presented treat the data as independent since, while participants agreed to be contacted in the future, they were not explicitly told they would be contacted again in 5 years and therefore were not expecting another interview. To examine how advance letters improved the response rates, the differences in the proportion between the groups was tested using Fisher’s Exact Test. A logistic regression was also completed to control for possible effects of the demographics since participants were not randomly assigned to be mailed a letter. Secondary analyses using non-parametric tests (variables were not normally distributed) were used to examine group differences with respect to resource use and expenses. All calculations were performed using IBM SPSS Statistics 24.0.

### Results

#### Demographics and smoking measures

Bivariate comparisons of the baseline demographics and smoking characteristics were made between the Letter and No Letter groups and, with the exception of “having previously used nicotine patches, gum, or inhalers in a previous quit attempt” (52.6% No Letter group, 68.3% Letter group; p = 0.011), no significant differences were observed (p > 0.05; Table 1).

**Table 1:** Baseline demographic characteristics between No Letter and Letter groups

Variable	Group^a^	
	No Letter (n = 138)	Letter (n = 137)
Age, mean years (SD)	47.5 (12.9)	48.7 (12.1)
Female sex	70 (50.7)	70 (51.1)
Married or common-law spouse	69 (50.0)	84 (61.3)
Employed full or part time	77 (56.2)	69 (50.4)
Education level		
Less than high school diploma	31 (22.5)	24 (17.5)
High school diploma	63 (45.7)	66 (48.2)
Postsecondary	44 (31.9)	47 (34.3)
Household income, $		
< 60,000	85 (64.4)	81 (62.3)
≥ 60,000	47 (35.6)	49 (37.7)
Cigarettes, mean (SD), days	18.3 (7.9)	18.4 (7.5)
FTND score, mean (SD)	4.8 (1.9)	5.1 (2.1)
Levels of nicotine dependence^c^		
Low	14 (10.4)	18 (13.4)
Low to moderate	46 (34.1)	33 (24.6)
Moderate	66 (48.9)	68 (50.7)
High	9 (6.7)	15 (11.2)
Age at first smoking, mean (SD), years	15.4 (4.8)	14.5 (4.2)
Time as smoker, mean (SD), years	23.1 (13.9)	25.0 (14.7)
No. of previous quit attempts		
0	5 (3.6)	11 (8.0)
1–5	95 (68.8)	89 (65.0)
> 6	38 (27.5)	37 (27.0)
Previously used NRT (patch, gum, inhaler) in a quit attempt	70 (52.6)	86 (68.3)^b^

*FTND* Fagerström Test for Nicotine Dependence; *NRT* nicotine replacement therapy

^a^Data are presented as number (percentage) of participants unless otherwise indicated. Sample sizes vary because of missing data on some variables

^b^*p* < 0.05

^c^Level of nicotine dependence is based on the FTND scores. Scores range from 1 to 10, with higher scores indicating a more intense physical dependence on nicotine. Low dependence corresponds to a score of 1 or 2, low to moderate dependence a score of 3 or 4, moderate dependence a score of 5 to 7, and high dependence a score of 8 to 10

#### Response and Refusal Rates

Response and refusal rates are presented in Table 2 [14]. While the response rate was higher and the refusal rate was lower in the Letter group, neither difference was statistically significant (p > 0.05; Table 2).

**Table 2:** Cooperation and refusal rates by subsample

Rate (%)	Group		*p*
	No Letter (n = 138)	Letter (n = 137)	
Response	69 (50.0)	81 (59.1)	0.147
Refusal	9 (6.5)	5 (3.6)	0.412

Since participants were not randomly assigned to receive a letter, a logistic regression was conducted to evaluate the effect of sending a letter on response rate, including baseline demographics (gender, language, age, marital status, employment status, education, income) as control variables. The model was significant (χ^2^(8) = 19.959, p = 0.010), explained 9.8% (Nagelkerke R^2^) of the variance, and correctly classified 62.8% of cases, however, the effect of the advance letter was not a significant predictor (p = 0.369) of response rate, thus the odds ratio was not interpreted.

#### Resource use and cost analysis

**Fig. 1:**
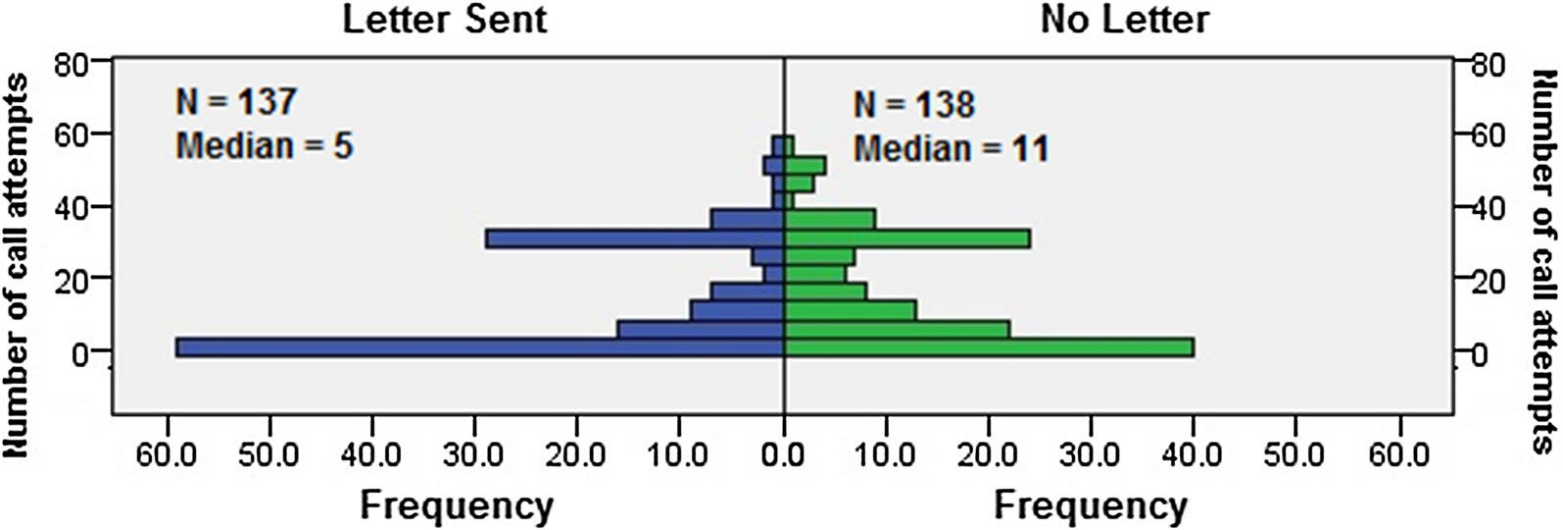
Distribution of call attempts between the Letter and No Letter groups

To determine if there was a difference in resource use, the number of call attempts per group was compared. Mann–Whitney U test was used since call attempts was not normally distributed and the assumption of equal distributions was satisfied through visual inspection. This revealed the distributions to be similar for the Letter and No Letter group (Fig. 1) confirming that the telephone approach described in the protocol was used consistently in both groups. The difference in the median number of call attempts for the Letter group (5 calls) and the No Letter group (11 calls) was significant (U = 7962.5, z = − 2.274, p = 0.023 two-tailed). This difference in median number of call attempts can be observed in Fig. 1.

Using estimates provided by the survey research company that conducted the telephone interviews, the cost of placing a telephone call that was not answered was CAD$0.60/call (average 70 calls/h, CAD$42.00/h). Similarly, the cost of preparing and sending the advance letter was estimated at CAD$2.99/letter [paper and printing (CAD$0.14), postage (CAD$1.00), and secretarial time (CAD$1.22)]. Mann–Whitney U test was used since costs was not normally distributed and visual inspection showed the distributions to similar in both groups, thus satisfying the assumption of equal distributions. Analysis showed a significant difference in the median cost between the Letter group (CAN$5.99) and the No Letter group (CAN$6.60) (U = 11,112.00, z = − 2.521, p = 0.012 two-tailed).

Another important cost was the amount spent on sending advanced letters in order to achieve one additional completed interview. Assuming the 9.1% difference in the response rates between the Letter and No Letter groups to be valid, 11 participants would have to be mailed an advance letter in order to have one additional interview completed (1/0.091 = 10.99). Mailing 11 letters would result in an additional cost of $32.89 per retained participant (11 × $2.99).

### Discussion

Mailing advanced letters to survey participants before attempting telephone contact is a common recommendation that is accepted as an easy, cost-effective method to increase response rates. This informed the decision to add an advance letter to our contact protocol approximately 4 months after starting follow-up interviews. This allowed us to compare a sample of participants who did and did not receive a letter.

Initial analysis of this nation-wide smoking cessation sample suggested that mailing an advance letter had an inconclusive effect on response rates (p > 0.05), even after controlling for demographics. However, further investigation of the impact of the letter on resource use demonstrated a significant six-call reduction in the median number of calls needed to reach participants who were sent an advance letter. While this was an important saving to resource use and personnel time, it was necessary to consider if this saving was offset by the additional CAD$2.99 cost of sending the letter.

A common criticism of studies involving advance letters is the tendency to not include a cost-analysis [8]. A significantly lower median cost of CAD$0.61 was found in the Letter group. Furthermore, the cost of retaining an additional participant was estimated at CAD$32.89 (per 11 participants). For this national study, an additional CAD$32.89 spent for the completion of an interview was considered worthwhile given the initial costs of recruitment, treatment, past interviews, and the high value placed on adequate follow-up rates in the context of a RCT. Thus, these results support the use of advance letters in studies, even though there was not a clear effect on the response rate.

The literature on the use of advance letters is extensive, but also contains contradictions [6]. While many studies report improvements in response rates, others, including this one, report smaller effects. While approaching significance, our findings do not clearly appear to support mailing an advance letter to improve the likelihood of reaching a participant. However, a significant saving in resource use and expenses, suggests that the advance letter may still add value to a protocol without directly effecting response. Further, the value of the overall response rate should be considered rather than only focusing on a significant increases at the p < 0.05 level. Indeed, the authors would argue that an absolute increase of less than that observed in the current study would be worthwhile given the importance of reducing attrition in RCTs.

Another factor that may contribute to the variable effects seen in the literature is the content of the letter. Our letter used several methods that have been previously studied and found to optimize participation [e.g. listing incentive ($30), social validation, reciprocity, authority, length of survey, length of letter] [8]. It would be difficult to determine which aspect influenced response rates most, but could be considered in future research. Until advance letters consistently increase follow-up rates, their merit and use in studies should still be evaluated. If survey participation continues to decline, ensuring high response rates will continue to be important, however, costs and effects on resources should be considered so research can be conducted efficiently and economically. It should not be assumed that any protocol change will offset costs, especially if postal rates continue to increase and telecommunication efficiencies continue to improve and present lower cost options.

## Limitations

- Participants were not randomly chosen to receive an advance letter.
- Telephone calls were completed by a well-established survey research firm that may have significantly reduced the costs/call.

## Additional file

**Additional file 1.** Sample Advance Letter. Sample of the text and letter format sent to participants before attempting contact by telephone.

## Abbreviations

CAD: Canadian Dollar
FTND: Fagerström Test for Nicotine Dependence
NRT: nicotine replacement therapy
RCT: randomized controlled trial

## References

[1] Dillman DA (2016). Moving survey methodology forward in our rapidly changing world: a commentary. J Rural Soc Sci.

[2] Kalton G (2000). Development in survey research in the past 25 years. Survey Methodol.

[3] Arfken CL, Balon R (2011). Declining participation in research studies. Psychother Psychosom.

[4] Galea S, Tracy M (2007). Participation rates in epidemiologic studies. Ann Epidemiol.

[5] Nathan G (2001). Telesurvey methodologies for household surveys - A review and some thoughts for the future. Survey Methodol.

[6] Sangster RL. Can we improve our methods to reduce nonresponse bias in RDD surveys? Am Stat Assoc. 2003:3642–3649.

[7] vanGelder MMHJ, Vlenterie R, IntHout J, Engelen LJLPG, Vrieling A (2018). Belt THvd: most response-inducing strategies do not increase participation in observational studies: a systematic review and meta-analysis. J Clin Epidemiol.

[8] de Leeuw E, Callegaro M, Hox J, Korendijk E, Lensvelt-Mulders G (2007). The influence of advance letters on response in telephone surveys: a meta-analysis. Public Opin Q.

[9] Carey RN, Reid A, Driscoll TR, Glass DC, Benke G, Fritschi L (2013). An advance letter did not increase the respone rates in a telephone survey: a randomized trial. J Clin Epidemiol.

[10] Snow RE, Prather JE, Hutcheson JD (1986). Program evaluation using a follow-up telephone survey: the effects of a prior letter. Eval Rev.

[11] Cunningham JA, Kushnir V, Selby P, Tyndale RF, Zawertailo L, Leatherdale ST (2016). Effect of mailing nicotine patches on tobacco cessation among adult smokers: a randomized clinical trial. JAMA Intern Med.

[12] Cunningham JA, Leatherdale ST, Selby PL, Tyndale RF, Zawertailo L, Kushnir V (2011). Randomized control trial of mailed nicotine replacement therapy to Canadian smokers: study protocol. BMC Public Health.

[13] Kushnir V, Selby P, Zawertailo L, Tyndale RF, Leatherdale ST, Cunningham JA (2018). Long-term effectiveness of mailed nicotine replacement therapy: study protocol of a randomized controll trial 5-year follow-up. BMC Public Health.

[14] Research AAoPO (2016). Standard definitions: final disposition codes and outcome rates for surveys.

