# Supplementary material for: To send or not to send: weighing the costs and benefits of mailing an advance letter to participants before a telephone survey
Source: BMC Res Notes. 2018 Nov 15;11:813. doi: 10.1186/s13104-018-3920-6 (PMC6238335; doi:10.1186/s13104-018-3920-6)
Supplement: Supplementary file 1 — Additional file 1. Sample Advance Letter. Sample of the text and letter format sent to participants before attempting contact by telephone. [file 13104_2018_3920_MOESM1_ESM.docx]

Additional File 1: Sample Advance Letter

[Date]

Dear [Participant Name],

About 4-5 years ago, you participated in a survey about smoking. Many thanks for your participation at that time. The study has been extended, and we are now following up with participants to ask some more questions about their smoking, or any changes in their smoking over the past 5 years.

Interviewers from the Survey Research Centre at the University of Waterloo will be calling in the next few weeks to ask if you would be willing to complete the survey. The survey will take approximately 15 minutes to complete. **Participants who complete the survey will be sent a $30 cheque.**

*If your phone number has changed, please contact the Survey Research Centre at [toll-free number].*

We would like to remind you that all information you provided will remain completely confidential. The results of this study will be reported so that you will not be identified and any published reports will refer to grouped information and not to any individual.

If you have questions about your participation or the research study, you can contact [Staff name], Project Coordinator, at the Centre for Addiction and Mental Health at [toll-free number, extension], or [staff email].

Sincerely,

Dr. John Cunningham

Senior Scientist

Centre for Addiction and Mental Health
